# From a voluntary vaccination policy to mandatory vaccination against COVID-19 in cancer patients: an empirical and interdisciplinary study in bioethics

**DOI:** 10.1186/s12910-022-00827-3

**Published:** 2022-08-28

**Authors:** Henri-Corto Stoeklé, Sakina Sekkate, Elisabeth Angellier, Titouan Kennel, Asmahane Benmaziane, May Mabro, Jean-François Geay, Philippe Beuzeboc, Christian Hervé

**Affiliations:** 1 Department of Ethics and Scientific Integrity, Foch Hospital, Suresnes, France; 2 Department of Oncology and Supportive Care, Foch Hospital, Suresnes, France; 3 Department of Supportive and Palliative Care, Institut Curie, Saint-Cloud, France; 4 Department of Clinical Research and Innovation, Foch Hospital, Suresnes, France; 5 Medical School, Paris Cité University, Paris, France; 6 Medical School, Versailles Saint-Quentin-en-Yvelines University, Montigny-le-Bretonneux, France; 7 Veterinary Academy of France, Paris, France; 8 International Academy of Medical Ethics and Public Health, Paris Cité University, Paris, France

**Keywords:** Pandemic, COVID-19, Cancer, Vaccine, Oncology, Bioethics, Hospital, France

## Abstract

**Background:**

At the start of 2021, oncologists lacked the necessary scientific knowledge to adapt their clinical practices optimally when faced with cancer patients refusing or reluctant to be vaccinated against COVID-19, despite the marked vulnerability of these patients to severe, and even fatal forms of this new viral infectious disease. Oncologists at Foch Hospital were confronted with this phenomenon, which was observed worldwide, in both the general population and the population of cancer patients.

**Methods:**

Between April and November 2021, the Ethics and Oncology Departments of Foch Hospital decided to investigate this subject, through an empirical and interdisciplinary study in bioethics. Our scientific objective was to try to identify and resolve the principal bio-ethical issues, with a view to improving clinical practices in oncology during future major pandemics of this kind, from a highly specific bio-ethical standpoint (= quality of life/survival). We used a mainly qualitative methodological approach based on questionnaires and interviews.

**Results:**

In April 2021, 29 cancer patients refused or were reluctant to be vaccinated (5.6%; 29/522). Seventeen of these patients said that making vaccination mandatory would have helped them to accept vaccination. In October 2021, only 10 cancer patients continued to maintain their refusal (1.9%; 10/522). One of the main reasons for the decrease in refusals was probably the introduction of the “*pass sanitaire*” (health pass) in July 2021, which rendered vaccination indispensable for many activities. However, even this was not sufficient to convince these 10 cancer patients.

**Conclusion:**

We identified a key bio-ethical issue, which we then tried to resolve: vaccination policy. We characterized a major tension between “the recommendation of anti-COVID-19 vaccination” (a new clinical practice) and “free will” (a moral value), and the duty to “protect each other” (a moral standard). Mandatory vaccination, at least in France, could resolve this tension, with positive effects on quality of life (i.e. happiness), or survival, in cancer patients initially refusing or reluctant to be vaccinated, but only if collective and individual scales are clearly distinguished.

## Background

At the start of 2021, during the progressive deployment of anti-COVID-19 (coronavirus disease 2019) vaccination in France, cancer patients were among those prioritized for vaccination because of their high vulnerability to severe, or even fatal forms of this new viral infectious disease [1–6]. However, oncologists, including those from the oncology department of Foch Hospital, were confronted with a refusal or reluctance of some of their cancer patients to be vaccinated against COVID-19. The Ethics and Oncology Departments of Foch Hospital therefore decided to perform an empirical and interdisciplinary study in bioethics to try to identify and resolve, qualitatively, the principal bio-ethical issues, with a view to concrete improvements in clinical practices in oncology during future major pandemics of this kind, from a very specific bioethical point of view.

## Methods

There are several theories in bioethics [7]. For various metabioethical reasons [7–10], we opted for a specific bioethical theory, adapted from the “global bioethics” (based on quality of life/survival) of the American bioethicist and biochemist Van Rensselaer Potter [11]. This theory contrasts strongly with the widely used theory of “principlism” (= autonomy, beneficence, non-maleficence, justice) of the American bioethicists and philosophers Tom Beauchamp and James Childress [12]. According to the bioethical theory adopted here, bioethics can also be seen as “*the science that studies new practices in life sciences *(*not only biomedical sciences*)* to try to identify and resolve* [bio-] *ethical issues *([i.e.] *tensions between* [moral] *values* [or] *standards and* [scientific or clinical] *practices*)* based on empirical research* [qualitative research, quantitative research, etc.]*, interdisciplinary studies *(*life sciences, human and social sciences, *etc*.*)* and inductive methods *(*probabilistic inference*)*, as well as actual or potential effects on quality of life* [i.e. happiness] *and/or the survival of the individuals and/or societies directly or indirectly concerned by these practices, and the social and complex phenomena that they constitute.*”[13]. We focused here on the quality of life (i.e. happiness) and survival of cancer patients refusing or reluctant to be vaccinated against COVID-19, at Foch Hospital, in France. Within the bioethical framework adopted here, we opted to use a specific action-research method, adapted from the work of the Canadian bioethicist and jurist Guy Durand (Table 1) [14].

**Table 1:** Action research method and appendix [14]

Key steps
*Step (1): Design*
→ State-of-the-art knowledge (= refusal rates, refusal characteristics, general population, cancer population) → Research problem & strategy (= main bioethical issues & qualitative approach)
*Step (2): Operationalization*
→ Target population(s) (= 29 cancer patients, 5 oncologists) → Methods of data collection & analysis (= questionnaires, non-directed interviews, semi-directed interviews & analysis of content)
*Step (3): Data collection & preparation*
→ Data collection (= telephone, consultations) → Data preparation (= Excel files, Word files, SAS v. 9.4 software)
*Step (4): Data analysis & interpretation*
→ Data analysis (= initial refusal rate: 5.6% i.e. 29/522 → final refusal rate: 1.9% i.e. 10/522) → Data interpretation (= vaccination policy, mandatory vaccination, collective *versus* individual scales, emergency MTM)

This study was approved by the institutional review board of Foch Hospital in France (IRB 00,012,437). Oral informed consent was obtained from all participants, with the approval of the same IRB. All methods were performed in accordance with the relevant guidelines and regulations in France [15, 16].

## Design

### State of the art

Within the general population [17–23], rates of vaccination hesitancy/refusal for COVID-19 have varied over space and time [24]. Indeed, in June 2021, the global rate of refusal was about 25%, with variation between countries. For example, the refusal rate was 48.4% in Russia, 43% in Nigeria, 40.7% in Poland, 20.8% in Canada, 18.8% in the United Kingdom and only 2.4% in China. The rate of refusal in France was 36.6%. [25].

However, the same reasons for hesitancy/refusal re-emerge time and again: expressions of doubt about the efficacy and safety of these vaccines [26], and about the dangerousness of the virus itself, from which a conspiracy-theory discourse may develop [27]. The individuals expressing such views are mostly women, young or poorly educated populations, with low incomes, often also reticent concerning anti-flu vaccination, living in rural areas and/or from ethnic minorities [28], such as the Afro-American population of the United States [29], or Arab populations in Israel [30].

The same markers have also been identified in cancer patients, together with several others specific to this population [24, 26–48], including, in particular, a belief that the vaccine will have a deleterious effect on their treatments [20, 21, 48], or that it might aggravate their disease [21], or even that it is simply contraindicated for cancer patients [17]. The global rate of refusal in the cancer patient population is lower than that for the general population, at about 10% [49], and the differences between countries are probably smaller than those for the general population. We were unable to find a precise rate for France.

Concerning the specific characteristics of this population, a Korean study reported that the cancer patients refusing or reluctant to be vaccinated tended to be those with recurrences of cancer, diagnosed less than five years ago and with a low EuroQol Visual Analog Scale score [18]. Evidently, in this population, being young (from 20 to just over 30 years, depending on the study) was not identified as a particular characteristic of those refusing or reluctant to be vaccinated, given that the median age at cancer diagnosis is 68 years in men and 67 years in women [50].

### Research problem and strategy

When we began to plan this study at the start of March 2021, very little was known on this subject, most of the studies cited above being published during the summer of 2021. For this reason, the Oncology and Ethics Departments of Foch Hospital decided to perform this study in collaboration. Our initial scientific objective (the research problem) was to identify and resolve the principal bio-ethical issues involved, so as to improve clinical practices in oncology specifically in the context of the COVID-19 pandemic. However, by the end of the study, we were focused more on improvements for future pandemics with similar systemic causes and effects. Retroactive approaches were difficult to achieve sufficiently rapidly and efficiently in the course of this pandemic, given the speed with which it developed.

We used a primarily qualitative approach, in which we focused on target populations. This methodological approach is, of course, subject to scientific limitations in terms of reproducibility, limiting the generalizability of our results.

## Operationalization

### Target populations

The Pfizer-BioNTech vaccine (an RNA vaccine) was offered to 522 patients treated for cancer at our hospital at the start of April 2021; 29 of these cancer patients refused or were reluctant to be vaccinated (a few other cancer patients were not or could not be included). These individuals formed the first target population, which we followed until November 2021. These 29 patients were characterized only in terms of their sex, type of tumor and cancer stage, to ensure that they could not be identified (Table 2). We then focused on the 10 cancer patients who continued to refuse vaccination in October 2021. Five different oncologists were broadly responsible for the management of these 10 cancer patients. These five oncologists constituted our second target population.

**Table 2:** Characteristics of the 29 cancer patients

Patients	**Patient 1**	Patient 2	Patient 3	Patient 4	**Patient 5**	Patient 6	**Patient 7**	**Patient 8**	Patient 9	**Patient 10**	Patient 11	Patient 12	**Patient 13**	Patient 14	Patient 15
*Characteristics*															
Sex	**Male**	Female	Male	Female	**Female**	Male	**Female**	**Male**	Female	**Female**	Male	Male	**Female**	Female	Female
Tumor	**Solid**	Solid	Solid	Solid	**Solid**	Solid	**Solid**	**Solid**	Solid	**Solid**	Solid	Solid	**Solid**	Solid	Solid
Stage	**Metastatic**	Metastatic	Metastatic	Metastatic	**Metastatic**	Metastatic	**Metastatic**	**Metastatic**	Non-metastatic	**Metastatic**	Non-metastatic	Metastatic	**Metastatic**	Non-metastatic	Metastatic

Patients	**Patient 16**	Patient 17	**Patient 18**	Patient 19	Patient 20	Patient 21	Patient 22	Patient 23	Patient 24	**Patient 25**	Patient 26	Patient 27	Patient 28	**Patient 29**
*Characteristics*														
Sex	**Female**	Female	**Female**	Female	Female	Female	Male	Female	Male	**Female**	Female	Female	Female	**Female**
Tumor	**Solid**	Solid	**Solid**	Solid	Solid	Solid	Solid	Solid	Solid	**Solid**	Solid	Solid	Solid	**Solid**
Stage	**Metastatic**	Metastatic	**Metastatic**	Metastatic	Metastatic	Metastatic	Metastatic	Non-metastatic	Non-metastatic	**Metastatic**	Metastatic	Non-metastatic	Metastatic	**Metastatic**

The 10 cancer patients refusing vaccination are highlighted in Bold

### Data collection and analysis methods

For the cancer patient target population, data were collected via questionnaires and non-directed interviews (Tables 1 and 3). The questionnaires were not anonymous and were administered to the 29 cancer patients refusing or reluctant to be vaccinated. The cancer patients were asked 11 questions in the month of April 2021, and then a single, identical question in the months of July and October 2021 (Tables 1 and 3). The questionnaires were mostly developed through a joint writing process involving the bioethicists of the Ethics Department and oncologists from the Oncology Department of Foch Hospital, in collaboration with another oncologist from the Curie Institute, in France. For the oncologist target population, data were collected through semi-directed interviews (Tables 1 and 4). The interview guide was written solely by the bioethicists of the Ethics Department.

**Table 3:** Questions asked of the cancer pacxstients at the various times points

*Questions—April 2021*
→ (1) Have you ever refused a vaccine for yourself or your children because you thought it was pointless or dangerous?
→ (2) Have you or your children already received a vaccine despite your doubts about its efficacy?
→ (3) Are your afraid of COVID-19?
→ (4) Do you know about the evaluations that have been performed for the vaccine you have been offered?
→ (5) Have you read any scientific documents on this subject?
→ (6) Have you seen or heard scientific information from doctors?
→ (7) Did it influence your decision?
→ (8) Have you read documents from other sources?
→ (9) Did that influence your decision?
→ (10) Do you trust “us” (i.e. the people treating your cancer)?
→ (11) Would it have made it easier for you to agree to be vaccinated if vaccination had been made obligatory?

*Question—July & October 2021*
→ Have you been vaccinated?

**Table 4:** Grids for the semi-directed interviews with oncologists

Semi-directed interview grid
→ (1) In one word, how would you define your human relationship with this patient?
→ (2) In your professional relationship, what line have you taken in your discussions about cancer with the patient?
→ (3) In response to what you said about the cancer, what was the reaction of the patient concerning the treatment proposed?
→ (4) Do you think the patient really understood?
→ (5) How did the attitude of the patient toward vaccination fit into this relationship?
→ (6) How would you define this patient relative to other patients?

With the exception of a few statistical analyses, the data collected were analyzed without the assistance of a computer. We performed an analysis of content for manual extraction of the pertinent information.

## Data collection and preparation

### Data collection

Questionnaire data for the cancer patient target population were collected by the oncologists treating the patients, during a consultation or telephone interview, in April, July and October 2021. Data were missing for certain cancer patients in April 2021. The data relating to the 10 cancer patients who continued to refuse vaccination in October 2021 were considered of particular scientific interest, so any missing data were recovered in November 2021, by the oncologists asking their patients to provide the necessary information directly, by telephone or during a consultation (for cancer patients 1, 13 and 29). For some cancer patients (cancer patients 9, 12, 21, 22, 23 and 26), we preferred not to ask the patients directly. Conditions changed between April and November 2021, so these data should be considered with great caution.

The non-directed interviews with these 10 cancer patients were carried out by a bioethicist, by telephone, in October 2021. These interviews were not recorded and were only partially retranscribed. The choice to perform these interviews by telephone and not to record them was motivated by a desire to avoid frightening these 10 cancer patients. In these conditions, it was difficult for the bioethicist to transcribe the entire conversation. Three cancer patients (cancer patients 1, 16 and 25) did not answer the telephone, and did not call back in response to a voicemail message. Again, we chose not to be too insistent, to avoid creating even more stress for these patients.

For the oncologist target population, data were obtained during a semi-directed interview performed by a bioethicist, at the hospital, in October 2021. It was possible to record these interviews and to retranscribe them in their entirety.

### Data preparation

The data collected by questionnaire were manually regrouped and structured in various Excel files. The data collected during the non-directed and semi-directed interviews were manually regrouped and structured in various Word files. All the final results were pseudo-anonymized.

A few statistical analyses were performed on the data for cancer patients from April 2021, by a biostatistician from the Department of Clinical Research and Innovation of Foch Hospital, with SAS v. 9.4 software. This quantitative approach remained secondary, due to the small size of our cancer patient target population (*n* = 29). These statistical analysis were based on frequencies and percentages for categorical variables. We used chi-squared or Fisher’s exact tests for the comparison of categorical variables. All tests were two-tailed, with an alpha risk of 5%.

## Analysis and interpretation

### Data analysis

All the cancer patients studied were managed at Foch Hospital for a solid tumor (breast, ovary, colon, pancreas, etc.), mostly at the metastatic stage (Table 2). The vaccine refusal rate among these patients in April 2021 was 5.6% (29/522).

The first key finding to emerge from the analysis of questionnaire results was that women were significantly more likely to refuse or to be reluctant to be vaccinated than men (*p* = 0.001) (Table 5). Indeed, 21 (9.6%; 21/218) of the 29 cancer patients refusing or reluctant to be vaccinated were women and eight were men (2.6%; 8/304), whereas there were more men (304) than women (218) in the total population of 522 cancer patients [6].

**Table 5:** Statistical analysis of COVID-19 vaccine refusal status by sex in April 2021

	Refusal (*N* = 29)	No refusal (*N* = 493)	*P* value
*Sex, N *(*%*)			
Female	21 (72.4)	197 (40.0)	0.001
Male	8 (27.6)	296 (60.0)	

The second key finding was that all 29 cancer patients said that they had never before refused vaccination, either for themselves or for their children (cancer patients 2, 6 and 23 had no children). However, three cancer patients had previously refused anti-flu vaccination (cancer patients 3, 15 and 16), which is not mandatory. Six of the cancer patients said that they had previously agreed to vaccination for themselves or for their children despite doubts about its efficacy (Table 6). Thus, none of these cancer patients appeared to have a particularly high degree of reticence concerning vaccination in general. We found no significant differences between the men and women in this population for this factor, except for question 6 (*p* = 0.03). However, this may be due to the relatively small sample size (*n* = 29). However, the refusal or reluctance observed here seemed to be clearly specific to vaccination against COVID-19.

**Table 6:** Responses of the 29 cancer patients in April 2021

Patients	**Patient 1**	Patient 2	Patient 3	Patient 4	**Patient 5**	Patient 6	**Patient 7**	**Patient 8**	Patient 9	**Patient 10**	Patient 11	Patient 12	**Patient 13**	Patient 14	Patient 15
*Questions/answers—April 2021*															
(1) Have you ever refused a vaccine for yourself or your children because you thought it was pointless or dangerous?	**No**	No	No	No	**No**	No	**No**	**No**	No	**No**	No	No	**No**	No	No
(2) Have you or your children already received a vaccine despite your doubts about its efficacy?	**No**	No	No	No	**No**	No	**No**	**No**	No	**No**	No	Yes	**Yes**	Yes	No
(3) Are you afraid of COVID-19?	**No**	No	No	No	**Yes**	No	**No**	**Yes**	Yes	**No**	No	Yes	**Yes**	No	Yes
(4) Do you know about the evaluations that have been performed for the vaccine you have been offered?	**Yes**	Yes	Yes	No	**No**	No	**No**	**Yes**	No answer	**No**	Yes	Yes	**Yes**	No	Yes
(5) Have you read any scientific documents on this subject?	**No**	No	No	Yes	**No**	No	**No**	**No**	No	**No**	Yes	No	**No**	No	No
(6) Have you seen or heard scientific information from doctors?	**Yes**	Yes	Yes	Yes	**Yes**	Yes	**No**	**Yes**	Yes	**No**	Yes	Yes	**Yes**	Yes	Yes
(7) Did it influence your decision?	No*	No	No	Yes	**No**	No	**No**	**Yes**	Yes	**No**	Yes	Yes	**Yes**	No	Yes
(8) Have you read documents from other sources?	**No**	No	No	No	**No**	Yes	**Yes**	**No**	Yes	**No**	Yes	No	**No**	No	No
(9) Did that influence your decision?	**No**	Yes	No	No	**No**	Yes	**No**	**No**	Yes	**No**	No	No answer	**No**	No	No
(10) Do you trust "us" (i.e. the people treating your cancer)?	**No**	Yes	Yes	Yes	**Yes**	Yes	**Yes**	**Yes**	Yes	**Yes**	Yes	Yes	**Yes**	Yes	Yes
(11) Would it have made it easier for you to agree to be vaccinated if vaccination had been made obligatory?	**No**	Yes	Yes	Yes	**No**	No	**No**	**Yes**	Yes	**Yes**	No	Yes	No*	Yes	No

Patients	**Patient 16**	Patient 17	**Patient 18**	Patient 19	Patient 20	Patient 21	Patient 22	Patient 23	Patient 24	**Patient 25**	Patient 26	Patient 27	Patient 28	**Patient 29**
*Questions/answers—April 2021*														
(1) Have you ever refused a vaccine for yourself or your children because you thought it was pointless or dangerous?	**No**	No	**No**	No	No	No	No	No	No	**No**	No	No	No	**No**
(2) Have you or your children already received a vaccine despite your doubts about its efficacy?	**No**	Yes	**Yes**	No	No	No	Yes	No	No	**No**	No	No	No	**No**
(3) Are you afraid of COVID-19?	**Yes**	No	**No**	No	No	Yes	No	No	No	**No**	Yes	No	Yes	**No**
(4) Do you know about the evaluations that have been performed for the vaccine you have been offered?	**No**	No	**No**	Yes	No	No	No	No answer	No	**No**	No	No	No	**No**
(5) Have you read any scientific documents on this subject?	**No**	No	**No**	No	No	No	No	No	No	**No**	No	No	No	**No**
(6) Have you seen or heard scientific information from doctors?	**No**	No	**No**	No	Yes	No	Yes	No	No	**No**	No	No	No	**No**
(7) Did it influence your decision?	**No**	No	**No**	Yes	Yes	No	Yes	No	No	**No**	No answer	No	No	**Yes**
(8) Have you read documents from other sources?	**Yes**	No	**No**	Yes	No	No	Yes	Yes	No	**No**	No	No	No	**Yes**
(9) Did that influence your decision?	**Yes**	No	**No**	Yes	Yes	No	Yes	Yes	No	**Non**	No	No	No	**Yes**
(10) Do you trust "us" (i.e. the people treating your cancer)?	**Yes**	Yes	**Yes**	Yes	Yes	Yes	Yes	Yes	Yes	**Yes**	Yes	Yes	Yes	Yes*
(11) Would it have made it easier for you to agree to be vaccinated if vaccination had been made obligatory?	**Yes**	Yes	**Yes**	No	No	No answer	No answer	Yes	No	**Yes**	Yes	Yes	Yes	**Yes**

The 10 cancer patients refusing vaccination are highlighted in Bold

*Responses missing in April 2021 but obtained in November 2021

The third key finding was that vaccine hesitancy/refusal was often associated with a relative lack of knowledge about the vaccine. Indeed, 10 cancer patients said that they were afraid of catching COVID-19 (Table 6). Cancer patient 20 responded that she had already had COVID-19. Nine cancer patients said that they knew about the evaluations performed on this new vaccine—cancer patients 9 and 23 did not answer this question—, and two cancer patients stated that they had read scientific documents on this subject (Table 6). Fifteen cancer patients said that they had received scientific information from oncologists, with 10 saying that this information had influenced their decisions (Table 6)—only at the beginning for cancer patient 15, and cancer patient 26 did not answer this question. Nine cancer patients said that they had read documents from other sources (media, social networks, internet, relatives, etc.) and that this had influenced their decision—cancer patient 12 did not answer this question (Table 6).

Two other results were of particular interest. Firstly, 28 cancer patients said that they had confidence in the oncologists responsible for managing their cancer (Table 6). Secondly, at least 17 cancer patients said that mandatory vaccination would have helped them to accept vaccination—cancer patients 21 and 22 did not answer this question (Table 6). It should be borne in mind that, at the time, vaccination against COVID-19 was mandatory only for healthcare workers (= doctors, midwives, nurses, etc.) and other professionals considered at risk (= firefighters, etc.) [51].

The July questionnaire revealed that eight cancer patients had agreed to be vaccinated despite their misgivings (Table 7). Four cancer patients (cancer patients 12, 20, 21 and 26) had died from their cancers between April and July 2021 (Table 7).

**Table 7:** Responses of the 29 cancer patients in July 2021

Patients	**Patient 1**	Patient 2	Patient 3	Patient 4	**Patient 5**	Patient 6	**Patient 7**	**Patient 8**	Patient 9	**Patient 10**	Patient 11	Patient 12	**Patient 13**	Patient 14	Patient 15
*Question/answers—July 2021*															
Have you been vaccinated?	**No**	Yes	No	Yes	**No**	No	**No**	**No**	Yes	**No**	Yes	Died	**No**	No	No

Patients	**Patient 16**	Patient 17	**Patient 18**	Patient 19	Patient 20	Patient 21	Patient 22	Patient 23	Patient 24	**Patient 25**	Patient 26	Patient 27	Patient 28	**Patient 29**
*Question/answers—July 2021*														
Have you been vaccinated?	**No**	No	**No**	Yes	Died	Died	No	Yes	Yes	**No**	Died	No	Yes	**No**

The 10 cancer patients refusing vaccination are highlighted in Bold

By October 2021, 15 cancer patients had agreed to be vaccinated, leaving only 10 cancer patients still refusing vaccination (cancer patients 1, 5, 7, 8, 10, 13, 16, 18, 25 and 29) (Table 8). Two of these cancer patients were men (cancer patients 1 and 8); the other eight were women (cancer patients 5, 7, 10, 13, 16, 18, 25 and 29), and all 15 had metastatic cancers. Six of these cancer patients (cancer patients 8, 10, 16, 18, 25 and 29) had indicated in April that mandatory vaccination would have helped them to accept vaccination (Tables 6 and 8).

**Table 8:** Responses of the 29 cancer patients in October 2021

Patients	**Patient 1**	Patient 2	Patient 3	Patient 4	**Patient 5**	Patient 6	**Patient 7**	**Patient 8**	Patient 9	**Patient 10**	Patient 11	Patient 12	**Patient 13**	Patient 14	Patient 15
*Question/answers—October 2021*															
Have you been vaccinated?	**No**	Yes	Yes	Yes	**No**	Yes	**No**	**No**	Yes	**No**	Yes	Died	**No**	Yes	Yes

Patients	**Patient 16**	Patient 17	**Patient 18**	Patient 19	Patient 20	Patient 21	Patient 22	Patient 23	Patient 24	**Patient 25**	Patient 26	Patient 27	Patient 28	**Patient 29**
*Question/answers—October 2021*														
Have you been vaccinated?	**No**	Yes	**Non**	Yes	Died	Died	Yes	Yes	Yes	**No**	Died	Yes	Yes	**No**

The 10 cancer patients refusing vaccination are highlighted in Bold

The data collected in the non-directed interviews revealed these 10 individuals to be very fragile, both physically and psychologically, due to their metastatic cancers, and terrified of the COVID-19 vaccine. Most of the seven people questioned (cancer patients 5, 7, 8, 10, 13, 18 and 29) were worried about the adverse effects of this vaccine and the risk that it might aggravate their fatigue (cancer patients 5, 7, 8, 13 and 18) or cause their death (cancer patient 29). Cancer patient 10 indicated a dislike of vaccines, despite being up-to-date for all the usual mandatory vaccines. As indicated above, the bioethicist was unable to contact cancer patients 1, 16 and 25. The non-directed interviews were relatively short (≤ 5 min).

The semi-directed interviews with the oncologists took longer (10–15 min). Five cancer patients (cancer patients 1, 10, 13, 18 and 25) were managed by the same oncologist (a woman), two cancer patients (cancer patients 7 and 29) were managed by another oncologist (a woman) and the remaining three cancer patients (cancer patients 5, 8 and 16) were managed by different oncologists (2 men and 1 woman). The oncologists’ responses were essentially convergent.

In response to question 1, “*In one word, how would you define your human relationship with this patient?*” (Table 4), certain oncologists considered that they had a relationship of “trust” with their cancer patients (cancer patients 1, 13, 18 and 25), whereas others had various difficulties (cancer patients 5, 7, 8 and 29). However, the response to question 6, “*How would you describe this patient relative to other patients?*”, was almost systematically negative, with the oncologists using words such as “*rebellious*” (cancer patients 1 and 25), “*reticent*” (cancer patient 10 and 13), “*manipulative*” (cancer patient 7) or “*atypical*” (cancer patient 5).

In response to question 2, “*In your professional relationship, what line have you taken in your discussions about cancer with the patient?*”, all the oncologists said that they had duly informed the cancer patient about a difficult prognosis, as all had metastatic cancers. The response to question 5, “*How did the attitude of the patient toward vaccination fit into this relationship?*”, was mostly “*fear of adverse effects*”.

The responses to questions 3 and 4 were less clear-cut (Table 4). In response to question 3, “*In response to what you said about the cancer, what was the reaction of the patient concerning the treatment proposed?*”, all five oncologists indicated that all the cancer patients followed their treatments, more or less rigorously (cancer patients 8 and 16), or that it had been difficult, to various degrees, to convince them to do so (cancer patients 5, 7 and 10). In response to question 4, “*Do you think the patient really understood?*” some of the oncologists felt that the cancer patients had not really understood the benefits of treatment (cancer patients 5 and 7). Moreover, by this time, cancer patient 6 was refusing to follow the anticancer treatment prescribed. The word “*denial*”, or a word of similar meaning, was used to describe the attitudes of cancer patients 1 and 29 to the incurability of their cancers.

It, thus, remained difficult to convince these 10 cancer patients to get themselves vaccinated against COVID-19. Just after the end of the study in November 2021, we learned that cancer patient 29 had finally agreed to be vaccinated, and that cancer patient 18 had been hospitalized with a severe form of COVID-19. These cancer patients were among those who had indicated in April that rendering vaccination obligatory would have made it easier to accept (Table 6). Cancer patient 1 died from cancer after the end of the study. As these events occurred after the end of the study, we considered all 10 cancer patients in the analysis.

### Data interpretation

In our view, the requirement for vaccination for many different activities imposed by the introduction of the “*pass sanitaire*”, on July 21, 2021 [52] was probably the trigger for 15 of the 29 cancer patients to agree to be vaccinated by October 2021 (Table 8). However, it is impossible to confirm this, with certainty, from our data alone, but it is clear that the introduction of the “*pass sanitaire*” in France led to a rapid large increase in the rate of vaccination against COVID-19 in the general population [52]. Moreover, according to prior knowledge, most of the characteristics of people refusing or hesitating to be vaccinated against COVID-19 in the general population were also present in the cancer patient population. It therefore seems highly likely that the introduction of the “*pass sanitaire*” had the same effect in both the cancer patient and general populations. However, the “*pass sanitaire*” does not appear to have been sufficient incentive for 10 cancer patients, despite five patients indicating in April 2021 that rendering vaccination mandatory would have helped them to accept COVID-19 vaccination (Table 6).

The recommendation of anti-COVID-19 vaccination initially created a strong tension with a major moral value (= to be), that of “free will” [53–56]. Indeed, cancer patient 7 clearly affirmed this value during the non-directed interview in October 2021, justifying the maintenance of refusal by saying “*we are free*”. This reason for refusal is espoused beyond the limits of Foch Hospital [57, 58]. In France, this value echoes the “*Loi relative aux droits des malades et à la qualité du système de santé*” (Law on the rights of patients and the quality of the health system) [59], which has, since March 2002, allowed any patient to refuse the heath care proposed. But can we really talk about “free will” when faced with something about which we have a very limited scientific knowledge, or low level of “scientific literacy”? Nothing is less sure [60]. According to our results (Table 6), all these cancer patients had a very limited scientific knowledge, or low level of “scientific literacy”, and knew little about vaccination practices. Ten of the 29 cancer patients probably continued to refuse vaccination not due to their knowledge, but due to fear.

This fear focused mainly on the adverse effects of the vaccine, and is entirely understandable. All of these patients with metastatic cancers (Table 2), were already having to cope with anticancer treatments that were relatively difficult to bear in terms of their adverse effects. They were very tired, both physically and psychologically, as highlighted above. It is understandable that, in such circumstances, collective considerations were not of primary importance to these cancer patients. In addition, their oncologists reported that these cancer patients had personalities that made them difficult to treat in general. This may well have been an important factor.

The recommendation of anti-COVID-19 vaccination also created another strong tension with a major moral standard (= to do): “protecting each other”. In this case, this moral standard implied a duty to be vaccinated not just through self-interest, but also for the benefit of others [53, 61–63]. This major moral standard seems to have had no significant impact on the 10 cancer patients who continued to refuse vaccination. There may be many diverse reasons for this, which probably pre-date the pandemic [64–66]. The alarmist epidemiological data that had recently been widely disseminated by the press and social networks were continuing to feed doubts about the efficacy of the vaccine against variants [67, 68]. However, these same epidemiological data demonstrated the efficacy of vaccination against the occurrence of serious, potentially lethal forms of COVID-19 and against disabling sequelae [68, 69].

From a strictly epidemiological standpoint, the collective benefits of anti-COVID-19 vaccination could be considered debatable, particularly with the predominance of the Delta variant [70]. However, it was clear that there was a direct and relatively large individual benefit of vaccination, particularly for people at risk, such as patients with cancer [1]. This brings us back, once again, to the question of mandatory vaccination.

From our bioethical point of view, mandatory vaccination was relatively recommendable in France. It may have been more debatable in other countries, particularly those with lesser roles of the state in the governance of society and individual health (e.g. North American countries) [71], or simply with poorly developed medical infrastructures hampering the optimal storage and circulation of certain anti-COVID-19 vaccines (e.g. sub-Saharan African countries) [70]. It remains clear that these new vaccines had a visible indirect positive effect on quality of life (happiness) and survival in the general population, at the individual and collective scales, thanks to their economic and social benefits [72]. Again, the “*pass sanitaire*”, which rendered vaccination essentially obligatory, at least if one wishes to live a “normal” life, has significantly increased the rate of vaccination in the general population in France, and probably in the population of cancer patients too. Nevertheless, mandatory vaccination, even in France, would imply several local adaptations, especially in hospitals.

Indeed, it seems unlikely that we would have been able to convince all of the remaining 10 cancer patients to get vaccinated, even if they were constrained to do so by law. Their personalities that made them difficult to treat generally. The serious incidents that occurred in Guadeloupe (France) at about the same time, involving the violent opposition of some French health workers to mandatory vaccination, tends to support this hypothesis [73]. But, above all, we feel that it would be debatable, bioethically, to impose the administration of a vaccine that, given the clinical profiles of these 10 cancer patients, might not necessarily improve their survival very much. Conversely, the anxiety generated by this vaccine may have had a non-negligible effect on their quality of life (i.e. happiness), not only due to their intrinsic personalities, but also due to the multiple physical, psychological and social consequences of their metastatic cancer and treatments. Given that patient 1 died of cancer just after the end of the study, it is probably no coincidence that he never called the bioethicist back. From our bioethical point of view, there, therefore, seems to be no major collective or individual value to be gained from obliging these few cancer patients to be vaccinated against COVID-19.

This did not mean doing nothing for these patients. On the contrary, it is important to act on a case-by-case basis, and collectively and actively, to assist the oncologists managing such cancer patients, if they so wish, through “emergency” multidisciplinary team meetings (MTM) [74]. We have been using MTM of this type at our hospital since the start of the pandemic, initially, during the first wave, to manage tensions linked to remote cancer patient management, but subsequently extended to the whole hospital to help prioritize vaccine doses in hospitals at the start of the vaccination campaign early in 2021 [75].

These emergency MTM were initially defined as “*a hybrid structure between multidisciplinary team meetings *[76]*, and the “ethical support cells” proposed by the CCNE* [French national ethics committee] [77, 78]*, which are also observed in other countries *[79]*. In the absence of serious scientific publications, these meetings are designed to collect a maximum of pertinent information relating to the pandemic, including, in particular, the first recommendations of public agencies or competent academic societies, and to use them to define, collectively, the first steps to be taken by the hospital, following an interdisciplinary analysis by team members including* [bioethicists and] *experts in human and social sciences. This should make it possible to decrease, as far as possible, the blind spots previously identified, which may have a strong negative effect on the quality of life and/or survival of the patients managed.*” [75]. With some adaptations to this new situation, such MTM could be entirely appropriate for the management of this small number of cancer patients resisting vaccination.

Furthermore, “*this approach extends the proposal of the French national ethics committee *(*CCNE*)* to create “ethical support cells” to help clinicians in cases of difficult medical decisions. However, this “emergency MTM” has a much stronger medical, scientific and reasoned dimension, making use of knowledge from life sciences and from human and social sciences.”* [80]. Perhaps the principal difference between these emergency MTM and other bioethics structures lies therein, with a much stronger focus on the strictly legal or philosophical dimensions of such topics. This might make these structures more acceptable to oncologists wary of bioethics structures. Of course, more empirical studies will be required to test this last hypothesis.

Finally, we should note the irritation of one of the oncologists, who found it “*difficult*” to “*accept the use of treatments that cost a fortune for a disease that is incurable, treatments that will have many more adverse effects […] rather than a vaccine*”. It should be borne in mind that in France, we have chosen to adopt, in addition to the state governance of health, a healthcare system allowing social solidarity and the collective management of a large proportion of medical costs via public health insurance, regardless of income [81]. The cancer patients continuing to refuse vaccination at the end of this study had benefited from the full reimbursement of treatments that are highly costly to the community (chemotherapy, immunotherapy, etc.) [82]. Even if they are highly vulnerable, this taking in hand of the costs of expensive cancer treatment should imply a certain individual and civic responsibility in line with the moral standard of “protecting each other”, because of the negative collective economic and social consequences of these individual choices, rather than an assertion of “free will”. It may also account for a certain irritation on the part of oncologists, and the general population, which should also be taken into account during emergency MTM, and could decrease feelings of guilt in some cases [74].

## Conclusion and perspectives

We identified and tried to resolve one main bio-ethical issue: vaccination policy. More precisely, according to our bioethical theory [13], and method (Table 1), we characterized a major tension between “the recommendation of anti-COVID-19 vaccination” (a new clinical practice) and “free will” (a moral value), but also the duty to “protect each other” (a moral standard). Mandatory vaccination could resolve this tension, at least in France, with positive effects on the quality of life (i.e. happiness), or survival of cancer patients refusing or reluctant to be vaccinated against COVID-19, but only if collective and individual scales are clearly distinguished.

Indeed, at the collective scale, mandatory vaccination of the general population during major pandemics of this kind may be an efficient means of limiting the number of cases of vaccine hesitancy/refusal. It would probably have a similar effect in the population of cancer patients, thereby enhancing the survival of these patients, given their greater vulnerability to severe and fatal forms of COVID-19. Nonetheless, further bioethical studies are required to investigate the dangers and risks of social discrimination and exclusion, particularly if the limitation of mandatory vaccination to particular at-risk populations, such as cancer patients, is considered.

At the individual scale, mandatory vaccination would be neither sufficient nor appropriate to convince cancer patients particularly strongly opposed to vaccination to change their minds, particularly for patients with metastatic cancer. The short-term survival of these individuals is already very uncertain, with or without COVID-19, and mandatory vaccination might increase their anxiety, thereby having a negative effect on the already low quality of life of these patients. Conversely, mandatory vaccination might have had a less negative effect on quality of life (i.e. happiness) in the patients who had finally agreed to be vaccinated by October 2021, perhaps even less negative than that for those who agreed to be vaccinated right at the start of the COVID-19 vaccination campaign. This question remains open.

Thus, even if mandatory vaccination is imposed at the collective scale, in the general population, and the timing and number of doses considered necessary determined scientifically, we should, at the individual scale, deal on a case-by-case basis with the very small number of cancer patients continuing to refuse vaccination, whilst collectively, and actively, assisting the oncologists responsible for their care, through a new kind of MTM, including both oncologists and bioethicists. The bio-ethical purpose of this MTM should be as follows: to find a pertinent balance between improving individual survival and individual quality of life (i.e. happiness), and collective imperatives, particularly in France, where the social security system collectively covers the cost of expensive cancer treatments.

Such MTM are likely to remain a one-off solution in the short and medium term. However, in the longer term, it will be important to improve communication between oncologists and cancer patients concerning new biotechnologies, including vaccines, with the help of bioethicists. It should be noted, however, that the overall rate of refusal in the cancer population (≈10%) [49] was lower than that in the general population (≈ 25%) [25], despite not necessarily being sufficient. This suggests that the population of cancer patients may have been globally better informed than the general population, or that cancer patients were aware of their greater vulnerability to this new potentially lethal infectious viral disease, and, thus, of the need for vaccination. Further empirical studies are also required to address this issue, and, more generally, more studies should be performed in other hospitals, and countries, especially those with different cultures, and on other diseases.

In conclusion, as pointed out above, we found it difficult to react rapidly and efficiently given the speed with which the COVID-19 pandemic progressed. Our findings should therefore be considered in the context of preparations for new major pandemics of this kind that may occur in the future [83]. Furthermore, our considerations are probably more relevant to countries with high-level medical resources, state governance of healthcare and public health insurance systems, like France.

## Data Availability

The datasets generated and/or analysed during the current study are not publicly available because they are entirely in French, but are available from the corresponding author on reasonable request.

## Abbreviations

CCNE: French national ethics committee
COVID-19: Coronavirus disease 2019
MTM: Multidisciplinary team meetings
IRB: Institutional review board
RNA: Ribonucleic acid
